# Blood pressure changes during tenofovir-based antiretroviral therapy among people living with HIV in Lilongwe, Malawi: results from the prospective LighTen Cohort Study

**DOI:** 10.1007/s00392-023-02253-w

**Published:** 2023-07-06

**Authors:** Hans-Michael Steffen, Melani Ratih Mahanani, Florian Neuhann, Angelina Nhlema, Philipp Kasper, Andrew de Forest, Thom Chaweza, Hannock Tweya, Tom Heller, Jane Chiwoko, Volker Winkler, Sam Phiri

**Affiliations:** 1 Department of Gastroenterology and Hepatology, University of Cologne, Faculty of Medicine and University Hospital Cologne, Cologne, Germany; 2 Hypertension Center, University of Cologne, Faculty of Medicine and University Hospital Cologne, Cologne, Germany; 3 Institute for Global Health, University of Heidelberg, Heidelberg, Germany; 4 School of Medicine and Clinical Sciences, Levy Mwanawasa Medical University, Lusaka, Zambia; 5 Lighthouse Clinic, Lilongwe, Malawi; 6 International Training and Education Center for Health, University of Washington, Seattle, WA USA; 7 Department of Global Health, University of Washington, Seattle, WA USA; 8 Department of Medicine, University of North Carolina School of Medicine, Chapel Hill, NC USA; 9 Department of Public Health and Family Medicine, Kamuzu University of Health Sciences, Lilongwe, Malawi

**Keywords:** HIV infection, Blood pressure, Weight gain, Hypertension prevalence, Hypertension incidence, Uncontrolled hypertension

## Abstract

**Background:**

Sub-Saharan Africa is one of the regions in the world with the highest numbers of uncontrolled hypertension as well as people living with HIV/AIDS (PLHIV). However, the association between hypertension and antiretroviral therapy is controversial.

**Methods:**

Participant demographics, medical history, laboratory values, WHO clinical stage, current medication, and anthropometric data were recorded at study entry and during study visits at 1, 3, 6 months, and every 6 months thereafter until month 36. Patients who stopped or changed their antiretroviral therapy (tenofovir, lamivudine, efavirenz) were censored on that day. Office blood pressure (BP) was categorized using ≥ 2 measurements on ≥ 2 occasions during the first three visits. Factors associated with systolic and mean BP were analyzed using bivariable and multivariable multilevel linear regression.

**Results:**

1,288 PLHIV (751 females, 58.3%) could be included and 832 completed the 36 months of observation. Weight gain and a higher BP level at study entry were associated with an increase in BP (*p* < 0.001), while female sex (*p* < 0.001), lower body weight at study entry (*p* < 0.001), and high glomerular filtration rate (*p* = 0.009) protected against a rise in BP. The rate of uncontrolled BP remained high (73.9% vs. 72.1%) and despite indication treatment, adjustments were realized in a minority of cases (13%).

**Conclusion:**

Adherence to antihypertensive treatment and weight control should be addressed in patient education programs at centers caring for PLHIV in low-resources settings like Malawi. Together with intensified training of medical staff to overcome provider inertia, improved control rates of hypertension might eventually be achieved.

**Trial registration:**

NCT02381275.

**Graphical abstract:**

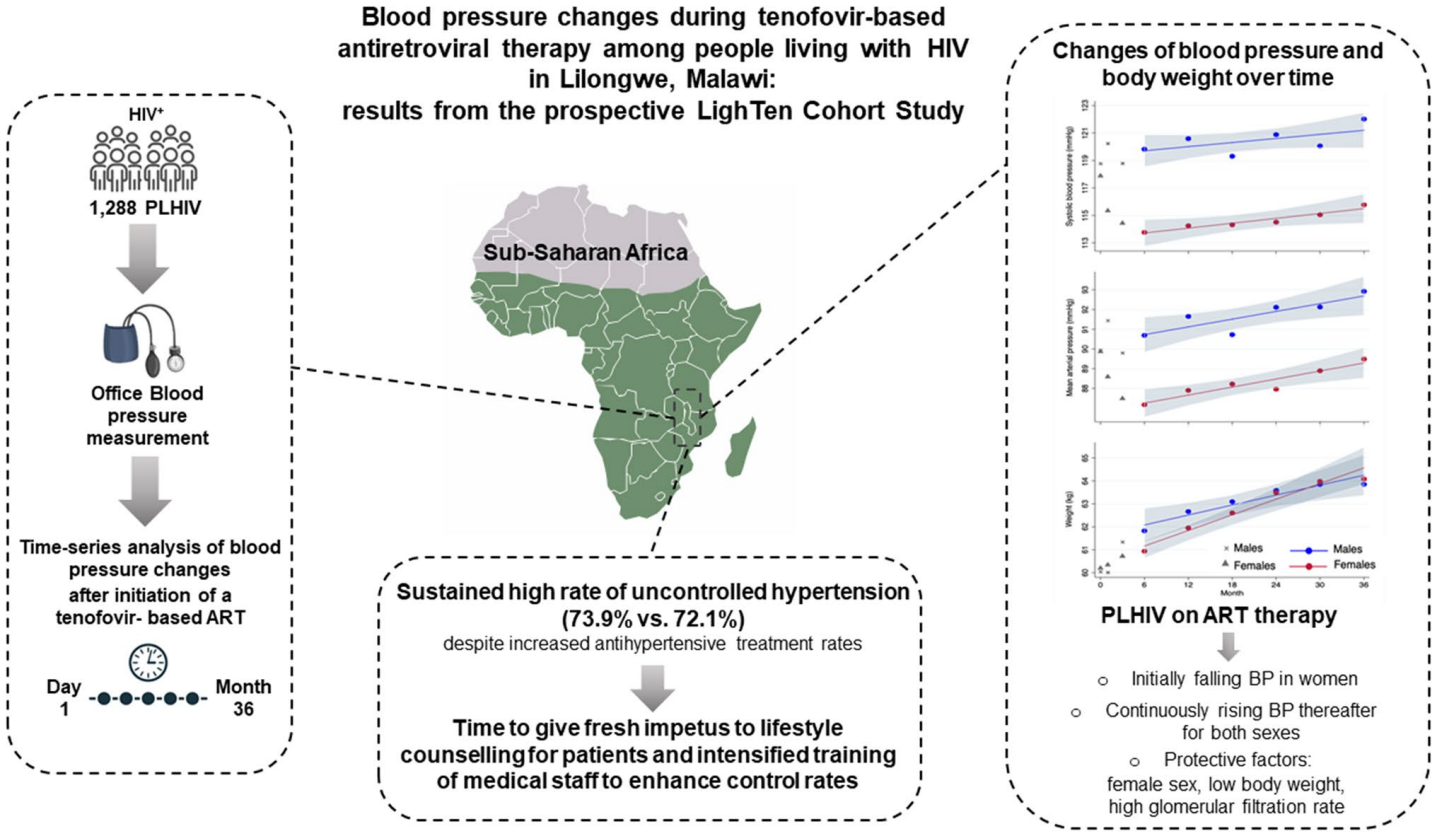

**Supplementary Information:**

The online version contains supplementary material available at 10.1007/s00392-023-02253-w.

## Introduction

The decades-long rise in cardiovascular disease (CVD) continues in almost all countries outside the high-income world and the CVD burden because of modifiable risk factors increases globally [1]. In 2019, the leading risk factor for attributable deaths was high systolic blood pressure (BP), which accounted for 10.8 million deaths, i.e., 19.2% of all deaths worldwide [2]. Three-quarters of the world population with hypertension are living in low- and middle-income countries [3]. Data on hypertension prevalence in Africa vary widely and depending on the age of the studied population range from 15 to 70% [4, 5]. Especially sub-Saharan Africa is one of the regions in the world with the highest fractions of uncontrolled BP at 91% in men and 87% in women, respectively [6].

Despite the progress made following the introduction of effective and widespread antiretroviral therapy (ART), this region still also has the highest global share of incident cases (64.8%), deaths (74.0%), and people living with HIV/AIDS (PLHIV, 70.7%) [7]. Improved survival on ART and sociodemographic changes have been accompanied by rising numbers of non-communicable diseases (NCD), especially CVD [8–10]. The HIV infection itself is associated with an elevated risk for CVD [11–13] and 20%–40% of PLHIV from the region, corresponding to 5–10 million individuals, are estimated to be hypertensive [14, 15].

Hypertension contributes considerably to the NCD burden, as hypertensive PLHIV are at a higher risk of cardiovascular events and suffer from increased all-cause mortality compared to hypertensive HIV-uninfected individuals or normotensive PLHIV [11–13, 16]. Pathophysiologic mechanisms in hypertensive PLHIV include a state of chronic systemic inflammation, immune dysregulation, viral tropism, microbial translocation [17, 18], and possibly direct ART effects, since the prevalence of hypertension has been found to be higher in PLHIV on ART (14.5%-35.0%) compared to those who were ART naïve (10.5–16.9%) [19–21].

The present analysis intends to contribute data from longitudinal observation to the discussion around the integration of NCD management into existing national HIV care programs in countries with limited resources [22]. To this end, we determined changes in BP and its determinants, prevalence, incidence, and control rate of arterial hypertension in ambulatory PLHIV who had initiated tenofovir-based ART in combination with lamivudine and efavirenz.

## Methods

The prospective Lighthouse Tenofovir (LighTen) Cohort Study included adult PLHIV presenting to the main facility of the Lighthouse Clinic located in Malawi’s capital city of Lilongwe for ART initiation. Recruitment started in August 2014 and follow-up ended in October 2019. As per Malawi HIV treatment guidelines at the time of enrollment [23], all patients on newly initiated ART received a fixed-dose combination of 300 mg tenofovir disoproxil fumarate (TDF), 300 mg lamivudine (3TC), and 600 mg efavirenz (EFV). The primary end point of the study was the change in renal function, while the prevalence and incidence of non-communicable co-morbidities including hypertension served as secondary objectives. The original power calculation was based on the expectation to detect a prevalence of 22% for renal impairment. However, due to technical reasons, creatinine and estimated glomerular filtration rate were available in a sufficient number of participants only at baseline. At the Lighthouse Clinic, the majority of routine clinical care is provided by nurses and clinical officers who complete 3–4 years of basic training.

### Study population

Adult patients (≥ 18 years) with confirmed HIV infection and no self-reported history of prior ART, willing to participate and able to give written informed consent, were included. Participant demographics and medical history including self-reported hypertension, smoking status, laboratory values, WHO clinical stage, current medication, and anthropometric data (height, body weight, body mass index (BMI)) were recorded at study entry (month 0) as well as during regular study visits at 1, 3, 6 months, and every 6 months thereafter until month 36. Weight was measured with shoes removed and only light clothing. Participants who stopped or changed ART regimen for whatever reason were censored on the date of treatment change as were the participants who withdrew their consent, were transferred to another HIV treatment center, or had missed two appointments or more. Following the guidelines, all participants received cotrimoxazole prophylaxis, while isoniazid preventive therapy was given only to a small minority of participants.

### Blood pressure measurements and definitions

Office BP was determined during each visit as the mean of the following measurements: (1) oscillometric BP measurement (Rossmax CF115f and Omron M300) as part of anthropometric measurements at clinical registration, and (2) additional measurement by a qualified nurse if the initial BP was ≥ 140/90 mmHg. All measurements were done in the sitting position 5 min after the cuff had been attached to the upper arm of the dominant hand. As a separate room for unattended BP measurements was not available, office BP was classified according to the current European Society of Cardiology/European Society of Hypertension treatment guidelines [24]. For the presentation of changes in BP categories from study entry to month 36, the classification of the 2020 International Society of Hypertension global hypertension practice guidelines with its lower number of categories was used [25]. Normotension was defined as mean BP < 140/90 mmHg per visit on at least two occasions during the first three visits (month 0, 1, and 3). As a mean BP ≥ 140/90 mmHg on one occasion does not label a patient as “hypertensive”, the individual median or modal BP category during the first three visits was considered as each participant’s baseline (month 0) BP category. Prevalent cases of hypertension were then defined as (i) the number of PLHIV with a median or modal BP category of at least hypertension grade 1 [24] plus (ii) participants on antihypertensive treatment, who were classified as “controlled “ or “uncontrolled” according to the aforementioned limits. Incident cases of hypertension were defined as mean systolic BP ≥ 140 mmHg or diastolic BP ≥ 90 mmHg on at least two consecutive visits from month 6 onward. Antihypertensive therapy was supposed to follow the Malawi Standard Treatment Guideline [26] and was initiated with lifestyle counselling (reduced intake of salt and alcohol, increased consumption of vegetables/fruit, combined with regular exercise and preferably normal body weight). Antihypertensive drug treatment was free of charge and prescribed depending on availability in a stepwise fashion (substances most frequently prescribed in square brackets) using a diuretic (hydrochlorothiazide [HCTZ] 25 mg qd, bendrofluazide 2.5 mg qd, furosemide 20 mg qd only in case of unavailability of other diuretics), a dihydropyridine calcium channel blocker (amlodipine [AML] 5-10 mg qd, nifedipine slow release 10-20 mg bid), an angiotensin-converting-enzyme inhibitor (enalapril [ENA] 10-20 mg qd, captopril 12.5-50 mg tid), and finally a betablocker (atenolol [ATEN] 50-100 mg qd, propranolol 40-80 mg tid). Angiotensin-receptor blockers or single pill combinations were not available during the study period.

### Laboratory analysis

All participants had a baseline evaluation beyond the standards of the Malawian HIV treatment program, including full blood count (AcT 5diff CP Hematology Analyzer, Beckman Coulter, Atlanta GA, USA), renal function tests (Erba XL200, Erba Mannheim, Germany), CD4 cell counts (Pima CD4-test, Abbott, Cape Town, South Africa), and HIV-RNA plasma levels (Cepheid GeneXpert, HIV viral load, Sunnyvale, CA, USA). The estimated glomerular filtration rate (eGFR) was calculated according to the CKD-EPI equation [27]. CD4 counts and viral load were measured every 6 and 12 months, respectively.

### Statistical analysis

Descriptive statistics included mean and standard deviation (for normally distributed variables), median and interquartile range (for non-normally distributed variables), and frequencies (%) for categorical variables.

Information on sex (binary; 0: female, 1: male), age (in years; continuous), months after enrollment (0 to 36; continuous), BP (systolic and diastolic; continuous), body weight and height (continuous), renal function parameter (eGFR according to the CKD-EPI equation, continuous), CD4 count (continuous), viral load (continuous), WHO HIV stage (categorical; 1, 2, 3, 4), and individual status (categorical; 1: alive on ART regimen not changed, 2: changed regimen, 3: lost to follow-up, 4: withdrawn, 5: died) were available for inductive analyses.

Systolic and mean arterial BP values ([2xdiastolic BP + systolic BP]/3) as main outcome variables were confirmed to be normally distributed using Kolmogorov–Smirnov’s test. Afterward, we examined the development of the variables with repeated measurements, i.e., systolic BP, mean arterial BP, and weight using scatter plots. As visual inspection revealed unsteadiness during the initial study period, linear regression was plotted from month 6 onward. Consequently, we assessed the factors associated with systolic and mean arterial BP also from month 6 onward, allowing repeated measurements of weight for individuals using bivariable and multivariable multilevel linear regression. Independent variables were months, sex, weight at each visit, BP at baseline, age at baseline, weight at baseline, log(eGFR) at baseline, and WHO HIV stage at baseline and the personal identifier (second level). Forward variable selection was performed to select the predictors for the multivariable multilevel models.

The BP classification were descriptively analyzed for the available 799 PLHIV being observed for the whole study period using Sankey diagrams comparing baseline and end line.

We compared participants enrolled at baseline vs. still enrolled at month 6 vs. not-enrolled patients seen at the Lighthouse Clinic during the recruitment period to check for selection bias. Missing values were treated as missing at random. We ran Wilcoxon rank-sum test (Mann–Whitney *U* test) to compare the median of viral load for the groups with controlled vs. uncontrolled hypertension at month 36. For the comparison of weight change for those who could vs. those who could not be followed until 36 months, independent t tests were used. All statistical analyses used 0.05 significance level.

All data analyses were performed using Stata/IC 15.1 for Mac (StataCorp LLC, 4905 Lakeway Drive, College Station, TX 77845, USA).

## Results

A total of 1,433 participants were enrolled during the 2-year recruitment period. At least two BP measurements on at least two occasions during the first 3 months were available for 1,288 PLHIV (751 females, 58.3%) who constitute the study population and the baseline characteristics are given in Table 1. The comparison of patients enrolled at baseline vs. month 6 vs. not-enrolled PLHIV revealed minor differences with respect to sex, age, BMI, and WHO stage at study entry (see Table 1 in the Supplementary Information).

**Table 1:** Characteristics at study entry for the whole study population and the subgroup observed until month 36

Characteristics	All observations			Visited month 36	
	*n*	%		*n*	%
Total	1,288	100.0		832	100.0
Female	751	58.3		488	58.7
Male	537	41.7		344	41.3
Age [years] (mean ± SD)	36.1 ± 9.3			37.1 ± 9.2	
Age group [years]					
18–24	117	9.1		57	6.8
25–34	503	39.1		302	36.3
35–44	445	34.5		307	36.9
45–54	170	13.2		128	15.4
55–64	43	3.3		33	4.0
65 +	10	0.8		5	0.6
Diabetes mellitus type 2	14	1.1		9	1.1
History of hypertension at study entry					
Self-reported, treated, uncontrolled	27	2.1		17	2.0
Self-reported, treated, controlled	10	0.8		5	0.6
Self-reported, untreated, uncontrolled	15	1.2		9	1.1
Self-reported, untreated, controlled	22	1.7		14	1.7
Systolic blood pressure [mmHg] (mean ± SD)	118.3 ± 20.4			119.9 ± 20.5	
Diastolic blood pressure [mmHg] (mean ± SD)	75.7 ± 14.0			76.6 ± 14.1	
Mean arterial pressure [mmHg] (mean ± SD)	89.9 ± 15.4			90.9 ± 15.5	
Blood pressure categories (ESC/ESH)					
Optimal	653	50.7		396	47.6
Normal	261	20.3		173	20.8
High normal	157	12.2		101	12.1
Grade 1 hypertension	154	12.0		117	14.1
Grade 2 hypertension	28	2.1		18	2.2
Grade 3 hypertension	35	2.7		27	3.2
Weight (at baseline) [kg] (mean ± SD)	60.1 ± 11.8			60.7 ± 12.0	
Missing	4	0.3		4	0.5
Body mass index [kg/m^2^]					
< 18.5	67	5.2		32	3.9
18.5–24.9	767	59.6		484	58.2
25.0–29.9	298	23.1		211	25.3
≥ 30.0	152	11.8		101	12.1
Missing	4	0.3		4	0.5
eGFR [mL/min/1.73m^2^] (median; IQR)	102; 84 – 116			101; 84 – 114	
Missing	12	0.9		6	0.7
WHO HIV stage					
1	603	46.8		403	48.4
2	212	16.4		147	17.7
3	396	30.8		237	28.5
4	77	6.0		45	5.4
CD4 count [cells/mm^3^] (median; IQR)	281; 128 – 426			296; 151 – 426	
Missing	182		14.1	149	17.9
Viral load [copies/mL] (median; IQR)	33,992; 6,968 –145,868			28,832; 6,012 – 129,634	
Missing	40		3.1	26	3.1
Hemoglobin [g/dL] (median; IQR)	12.5; 11.0 – 13.8			12.6; 11.0 – 13.9	
Missing^a^	236		18.3	139	16.7
Hematocrit [%] (median; IQR)	37.7; 33.1 – 41.9			38.1; 33.4 – 42.3	
RBC [10^6^/mm^3^] (median; IQR)	4.40; 3.96 – 4.88			4.42; 4.00 – 4.92	
WBC [cells/mm^3^] (median; IQR)	4,500; 3,600 –5,600			4,450; 3,600 – 5,500	
Platelet count [10^3^/mm^3^] (median; IQR)	226; 174 – 295			222; 172 – 286	
Missing	363		28.2	224	26.9

*SD* standard deviation, *IQR* interquartile range, *eGFR* estimated glomerular filtration rate, *RBC* erythrocyte count, *WBC* leucocyte count, ^a^applies also to hematocrit, RBC, WBC

**Fig. 1:**
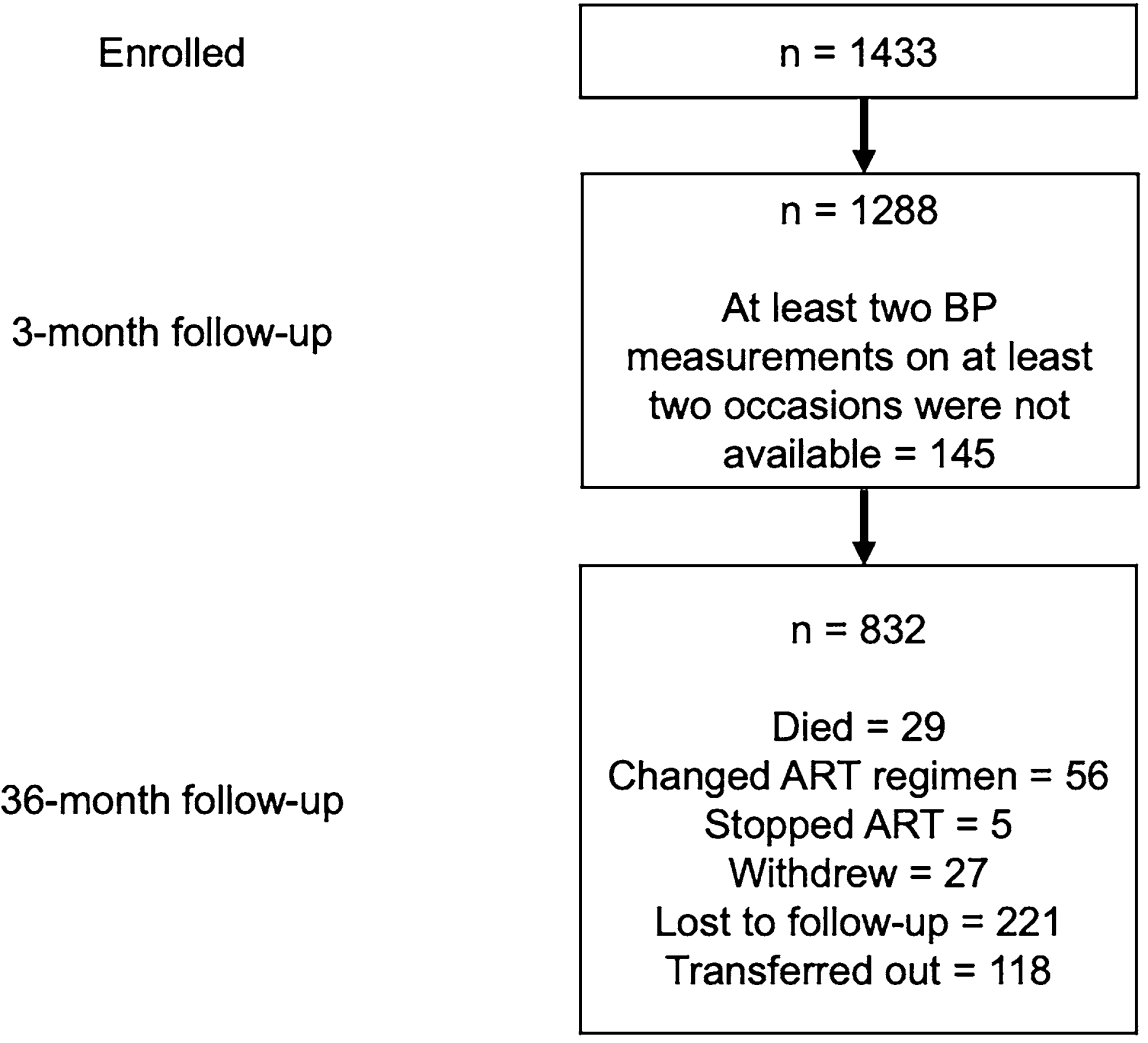
LighTen Study flowchart

During the observation time, a total of 456 participants were lost (see Fig. 1) including 29 participants who died, 56 patients who had their ART regimen changed due to failing viral suppression, and five PLHIV who had decided of their own free will to stop ART completely (for more details see Table 2 in the Supplementary Information). HIV plasma levels were below the detection limit (indicating ART drug adherence) in 96.0%, 98.0%, and 100% of females as well as 94.2%, 93.0%, and 97.1% of males at months 12, 24, and 36, respectively.

**Table 2:** Bivariable multilevel linear regression (≥ 6 months) for systolic and mean arterial BP

Variable	Systolic BP		Mean arterial BP	
	*ß*	*p* value	*ß*	*p* value
Months	0.1	0.018	0.1	< 0.001
Constant	116.0	< 0.001	88.3	< 0.001
Systolic BP at baseline	0.4	< 0.001	–	–
Mean arterial BP at baseline	–	–	0.4	< 0.001
Constant	67.6	< 0.001	50.9	< 0.001
Age at baseline	0.5	< 0.001	0.4	< 0.001
Constant	98.8	< 0.001	76.4	< 0.001
Sex				
Females	Ref	< 0.001	Ref	< 0.001
Males	5.5		3.2	
Constant	114.3	< 0.001	88.0	< 0.001
Weight at baseline	0.4	< 0.001	0.3	< 0.001
Constant	93.2	< 0.001	71.4	< 0.001
Weight at each visit	0.5	< 0.001	0.3	< 0.001
Constant	86.7	< 0.001	67.7	< 0.001
log(eGFR) at baseline	− 10.9	< 0.001	− 7.8	< 0.001
Constant	166.3	< 0.001	125.1	< 0.001
WHO HIV stage				
1	Ref	0.604	Ref	0.495
2	− 1.1		− 0.8	
3	0.7		0.6	
4	− 0.5		− 0.6	
Constant	116.6	< 0.001	89.3	< 0.001
CD4 count at baseline	− 0.1	0.816	0.1	0.795
Constant	117.1	< 0.001	88.8	< 0.001

*Months* number of months after the baseline visit, *BP* blood pressure, *eGFR* estimated glomerular filtration rate

There was a drop in both systolic and mean arterial BP during the first 6 months among female participants. Systolic and mean BP values were higher among males and both main outcomes as well as weight at each visit showed a linear increase with an almost identical slope for both sexes after month 6 (see figure in the Graphical Abstract). The change in weight (weight at last visit – weight at month 6) was correlated with the change in systolic (*r* = 0.212; *p* < 0.001) and mean (*r* = 0.194; *p* < 0.001) arterial BP (see Fig. 1 and Fig. 2 in the Supplementary Information).

**Fig. 2:**
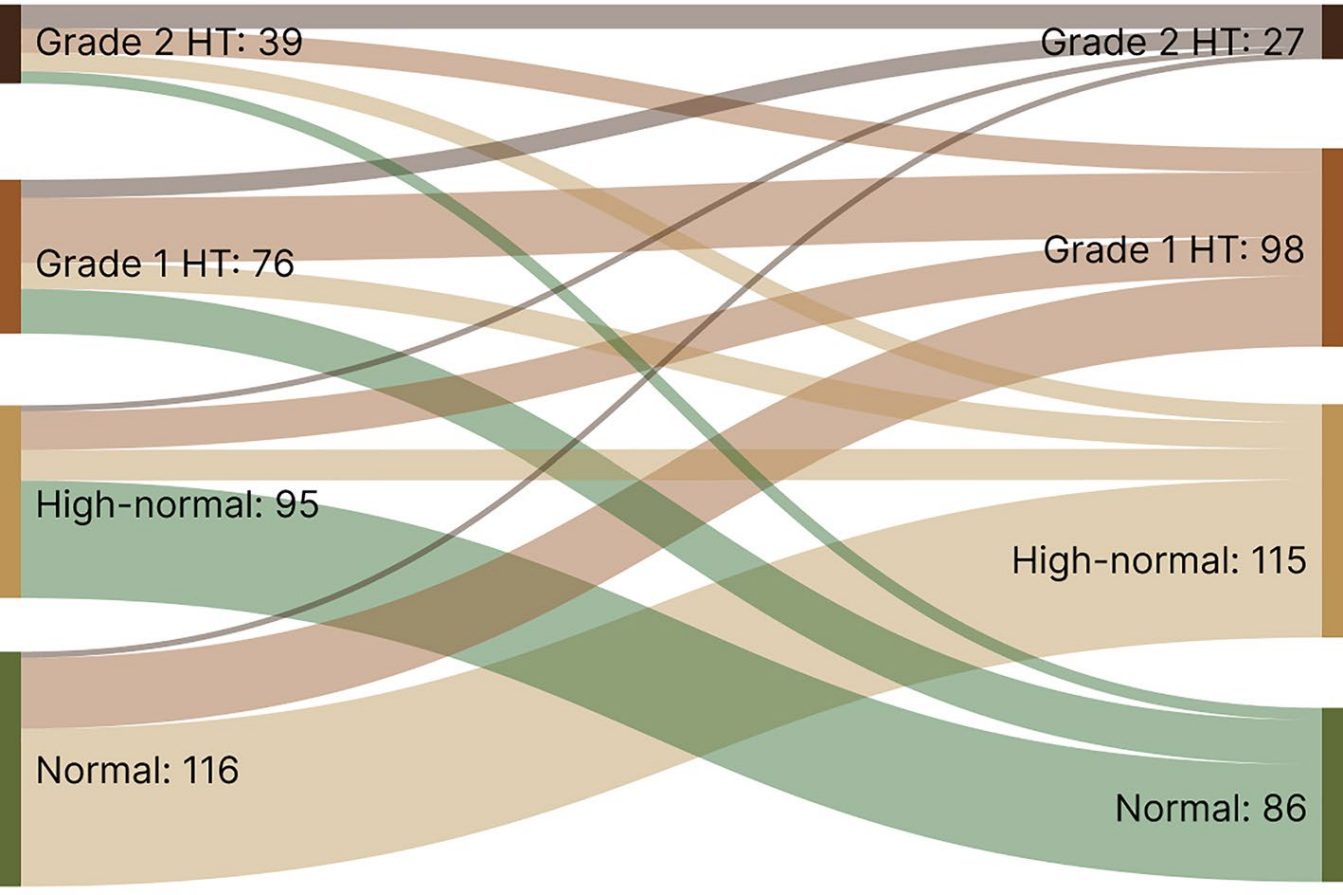
Blood pressure classification development over time (month 0 & 36) according to International Society of Hypertension categories. 473 (80.3%) unchanged observations of normotension are not shown in this figure, (https://sankeymatic.com/build/)

In bivariable analyses, sex, months (i.e., number of months after the baseline visit), and weight at each visit as well as baseline values for BP, age, weight, and log(eGFR) were associated with systolic as well as with mean arterial BP (Table 2). Multivariable analyses yielded similar results for both main outcomes and if stratified by sex (Table 3 and Table 4). In comparison to the bivariable analyses, the adjusted models showed smaller estimates for age at baseline, sex, and log(eGFR) at baseline, while higher weight at baseline changed to a negative effect. Weight at each visit resulted in similar estimates for all models showing an increase over time.

**Table 3:** Multivariable multilevel linear regression (≥ 6 months) for systolic BP stratified by sex

Variable	Both sexes		Females		Males	
	*ß*	*p*-value	*ß*	*p*-value	*ß*	*p*-value
Months	− 0.1	< 0.001	− 0.1	0.081	− 0.1	0.001
Systolic BP at baseline	0.4	< 0.001	0.4	< 0.001	0.3	< 0.001
Age at baseline	0.1	0.004	0.1	0.007	0.1	0.201
Sex						
Females	Ref	< 0.001	–	–	–	–
Males	4.6		–	–	–	–
Weight at baseline	− 0.4	< 0.001	− 0.4	< 0.001	− 0.5	< 0.001
Weight at each visit	0.6	< 0.001	0.5	< 0.001	0.8	< 0.001
log(eGFR) at baseline	− 6.2	< 0.001	− 7.3	< 0.001	− 4.8	0.008
Constant	81.4	< 0.001	83.6	< 0.001	83.1	< 0.001

*Months* number of months after the baseline visit, *BP* blood pressure, *eGFR* estimated glomerular filtration rate

**Table 4:** Multivariable multilevel linear regression (≥ 6 months) for mean arterial BP stratified by sex

Variable	Both sexes		Females		Males	
	ß	*p*-value	*ß*	*p*-value	*ß*	*p*-value
Months	0.1	0.866	0.1	0.318	− 0.1	0.468
Mean arterial BP at baseline	0.4	< 0.001	0.4	< 0.001	0.4	< 0.001
Age at baseline	0.1	0.009	0.1	0.023	0.1	0.126
Sex						
Females	Ref	< 0.001	–	–	–	–
Males	2.8		–	–	–	–
Weight at baseline	− 0.3	< 0.001	− 0.2	< 0.001	− 0.3	< 0.001
Weight at each visit	0.4	< 0.001	0.3	< 0.001	0.5	< 0.001
log(eGFR) at baseline	− 4.5	< 0.001	− 4.5	< 0.001	− 3.9	0.004
Constant	60.5	< 0.001	59.9	< 0.001	60.8	< 0.001

*Months* number of months after the baseline visit, *BP* blood pressure, *eGFR* estimated glomerular filtration rate

At study entry (month 0), a BP ≥ 140/90 mmHg was found in 217 participants (16.8%) and 74 PLHIV reported a history of hypertension, of whom 37 were not taking antihypertensive drugs. BP values were always < 140/90 mmHg during the first three visits in 22 of these 37 PLHIV with self-reported, but untreated hypertension, hence they were defined as normotensive. The remaining 52 participants were considered as truly hypertensive and together with newly detected cases (n = 121) the prevalence of PLHIV with confirmed hypertension was 13.4% for the whole group at study entry (11.5% for females and 16.2% for males). During the observation time, 50 PLHIV normotensive at study entry were diagnosed with hypertension for the first time, resulting in an incidence of 4.5% over up to 36 months. The number of prevalent and incident cases of hypertension was higher among male participants, but there were also more males in the older age groups (Table 5).

**Table 5:** Prevalent and incident cases of hypertension in female and male participants

Age group [years]	Females			Males			Both sexes
	Prevalent cases [*n*]	Incident cases [*n*]	Total female [*n*]	Prevalent cases [n]	Incident cases [n]	Total male [*n*]	
18–24	4	1	96	1	0	21	117
25–34	13	7	322	11	8	181	503
35–44	30	9	225	40	12	220	445
45–54	29	7	89	21	4	81	170
55–64	8	0	16	12	1	27	43
65 +	2	0	3	2	1	7	10
Total	86	24	751	87	26	537	1288

The change in the distribution of BP categories from baseline to month 36 is shown in Fig. 2 except for those whose BP remained normal (*n* = 473). Among these participants, the total number of PLHIV with a confirmed diagnosis of hypertension increased from 121 (15.1%) to 142 (17.8%). The number of patients on antihypertensive drug treatment nearly tripled (from 23 to 61); however, the rate of uncontrolled treated hypertension remained essentially unchanged: 17/23 PLHIV (73.9%) at study entry vs. 44/61 patients (72.1%) at month 36. Viral load at baseline did not differ between participants who finally had controlled vs. uncontrolled hypertension (28,832; 6,775–137,957 vs. 41,426; 7,794–149,943; *p *= 0.113). HIV replication was unsuppressed in one patient with controlled hypertension (on antihypertensive drug treatment) compared to three patients with uncontrolled hypertension (following lifestyle advice, but not on drug treatment). During the study, most hypertensive patients received antihypertensive monotherapy (*n* = 46, 85% on HCTZ), followed by free combinations of two (*n* = 24, 63% on ENA plus HCTZ), three (*n* = 10, 70% on ENA plus HCTZ plus AML), and four drugs, respectively (*n* = 3, ENA, HCTZ, AML plus ATEN). During 1,462 visits, modifications of antihypertensive drug regimens would have been indicated in 576 cases according to current guideline recommendations; however, the treating clinical officers and nurses adjusted the antihypertensive treatment during only 75 visits (13%).

## Discussion

The principal findings of the present prospective cohort study are sex-related differences in BP change during the first 6 months, followed by continuously rising BP thereafter in PLHIV who had started tenofovir-based ART. Weight gain during follow-up and a higher BP level at study entry favored a rising BP, while a negative association was found with female sex, lower body weight, and high glomerular filtration rate at baseline. Finally, despite an increase in antihypertensive drug prescriptions, the rate of uncontrolled hypertension remained essentially unchanged.

Pathophysiologic mechanisms in hypertensive PLHIV include a state of chronic systemic inflammation, immune dysregulation, viral tropism, and microbial translocation [17, 18] as well as HIV-mediated activation of the sympathetic nervous system [28, 29]. Neuroendocrine hyperactivity and autonomic dysfunction may be partially explained by psychosocial stressors [30], especially among PLHIV from low- and-middle income countries who often live at the minimum level of subsistence. Direct ART effects may also play a role, since the prevalence of hypertension has been found to be higher in PLHIV on ART (14.5%-35.0%) compared to those who were ART naïve (10.5–16.9%) [19–21]. The latter is comparable to the 16.8% in the present study, when only the office BP measurements from the first visit (month 0) were analyzed. In a large cross-sectional, population-based study in rural and urban Malawi using BP data from BP measurements of only one visit [31], the national prevalence was 15.8%. However, it is well known that the prevalence of hypertension is overestimated when office BP data from only a single visit are used [32]. In the present study, the number of hypertensive cases was lower (13.4%), once several BP measurements over several weeks were considered as requested by international guidelines to confirm a diagnosis of hypertension [24, 25, 33]. As could be expected, the percentage of hypertensive postmenopausal women was higher than men from the same age groups [34]. The observed significant sex difference in the overall hypertension prevalence (11.5% in females vs. 16.2% in males; p < 0.001) in the LighTen Study might be explained by the fact that more than 85% of female PLHIV were premenopausal.

There are already several investigations addressing weight gain on ART [35–41], and existing studies estimate the weight gain on EFV- and TDF-containing regimens to be moderate, in the range of 2–3 kg over 96 weeks [35, 42]. In a prospective study from South Africa ART with TDF, FTC, and efavirenz, a weight gain of 1 kg had been observed after 48 weeks [42]. The increase in body weight among LighTen participants is comparable to these findings.

According to a meta-analysis including more than 2.3 million participants, the risk of hypertension increased continuously with increasing BMI or weight gain [43]. Cross-sectional studies have confirmed overweight or obesity as determinants of arterial hypertension in countries from sub-Saharan Africa including Malawi, in PLHIV [44–47]. The hypertension incidence per 100 patient-years in prospective studies of PLHIV from sub-Saharan Africa ranges from 5.4 to 16.0, respectively [48–52]. This large variation may be explained by methodological differences related to the technique of BP measurement. Thus, the lower rate in the study from South Africa [50] was based upon three BP measurements on at least two occasions, while the highest rate was obtained in Ethiopia from two measurements on only one occasion [48], emphasizing the need to follow the international guidelines for an accurate diagnosis of hypertension [24, 25, 33]. In contrast to the LighTen Study, patients received a wide range of different combinations of NNRTIs and NRTIs [49–52] in these studies, but also DTG-based ART regimen [48]. Kidney disease with impaired eGFR is strongly associated with hypertension in PLHIV [50, 51, 53], which in turn may explain why high eGFR at study entry was found to have the strongest protective impact in LighTen participants.

According to a systematic review with meta-analysis, less than 40% of hypertensive individuals in the general population of sub-Saharan Africa were diagnosed as such, less than 20% of those diagnosed received antihypertensive medication and in less than 10% of treated individuals BP was controlled, i.e., < 140/90 mmHg [4]. A similar pattern can be observed among hypertensive PLHIV from sub-Saharan Africa, where the rate of uncontrolled hypertension is very high, ranging from 61 to 85%, and the rates of hypertension awareness are typically < 30% [54–57]. According to previous findings from an approach integrating hypertension management into HIV care facilities in Malawi, the BP control rates after 6 months were 38% and 30% among PLHIV with hypertension grade 1 or 2, respectively [57], which is comparable to a control rate of 37% for those on antihypertensive drug treatment in the general population [28]. Studies using an intensified model of care with visits every 6 weeks until BP was controlled led to higher control rates of 47.4% [58]. However, a recently published study of Malawian PLHIV with a median age of 51.0 years who were on ART for a median duration of 5.9 years (78.5% on efavirenz/lamivudine/tenofovir) revealed a control rate of only 19% after 1 year of follow-up for those on antihypertensive drug treatment [59]. The authors identified self-reported non-adherence to BP lowering medications as the only factor significantly associated with uncontrolled hypertension in this patient cohort with > 95% viral suppression [59]. Besides patient-related factors such as non-adherence, the reasons for uncontrolled BP are manifold, including irregular supply of antihypertensive drugs with frequent changes in prescriptions (and hence reduced adherence), substandard quality of drugs, shortage of health workers and centers, and poverty in general [60, 61]. In addition, physician inertia is a well-known phenomenon in the case of treated, but uncontrolled hypertension [62]. The same phenomenon has been observed for treating clinical officers and nurses in the present LighTen Study with its low rate (13%) of treatment adjustments in patients with BP ≥ 140/90 mmHg during patient visits.

In sub-Saharan Africa, girls and young women are twice as likely to be living with HIV than men [63]. The age distribution in the current study from Malawi mirrors this observation from epidemiological surveys of the region. Systolic and mean arterial BP were nearly identical for male and female participants when entering the study, but a stepwise decrease in BP over the next 6 months was observed in female PLHIV. Most patients (> 90%) were recruited on the same day as the diagnosis of HIV infection was confirmed. For women, the confirmed diagnosis might be more stressful with increased anxiety, as the fear of stigma, discrimination, abandonment, partner violence, and economic pressure in raising their children are more prevalent among women in Africa [64–68]. Also, a white coat effect, which is associated with anxiety [69] and more common among women [70, 71], may have been attenuated with repeated visits [72], hence explaining the observed sex-related differences during the first 6 months.

Systolic and diastolic BP independently predict adverse cardiovascular outcomes, although systolic BP has a greater effect [73], especially in older patients [74], while MAP is probably a better predictor in people aged < 60 years [75]. Also, MAP had been used to analyze the decline in renal function in patients with chronic kidney disease [76] and was recently found to have the highest predictability in detecting hypertension-related cerebrovascular alterations compared to systolic or diastolic BP separately [77].

According to current European and American guidelines BP should be measured in both arms at the first visit and to use the arm with the higher value as reference to avoid misclassification of BP [24, 33]. Difficulties to measure BP in both arms in a busy outpatient department may arise due to lack of time and workforce; thus, the ISH deemed bilateral measurements not essential for low- and middle-income countries ISH [25]. In a large cross-sectional, population-based study in rural and urban Malawi, measurements always in the right arm were used [31] in contrast to the WHO Steps survey, where BP measurements had to be taken always in the left arm [78]. Inter-arm pressure differences are inconsistent [79] and often not reproducible [80]. The BP is typically higher in the right arm [79, 81] and this has been explained by the handedness of people: right arm systolic BP ≥ 5 mmHg higher than left arm in 64% of right-handed participants (1,730 of 2,692 healthy volunteers) and left arm systolic BP ≥ 5 mmHg higher than right arm in 62% of left-handed participants (211 of 338 healthy volunteers) with differences < 5 mmHg in 30% and 35%, respectively [82]. To avoid shifting measurement sites, BP measurements were always taken in the upper arm of the dominant hand of the LighTen participants, thereby reducing variability in the individual BP change over time. In view of the above-mentioned inter-arm differences, the number of cases whose BP category would differ due to this type of measurement seems negligible.

## Strengths and limitations

The results of the LighTen Study are based on findings from a well-defined population with regular follow-up visits. Patients were included in the analysis as long as they received the same antiretroviral drug combination with TDF, FTC, and efavirenz they had initiated at study entry. No major differences were identified compared to non-enrolled PLHIV seen at the Lighthouse through the recruitment period. 65% of the study cohort completed the 36 months visit; thus, a third of patients has been lost to follow-up, mainly because they had defaulted two or more scheduled visits or had been transferred out to other HIV treatment centers. Again, compared to the whole cohort, no major differences in demographic data were identified. The initial weight gain for those who did not complete the study was not different from those who could be followed until 36 months (see Table 3 in the Supplementary Information). However, less increase after month 6 and finally a weight loss were observed for the former group. Patients who had died or needed a change in ART due to viral resistance had been included in this group. Thus, it cannot be excluded that the study findings are biased toward a “healthier” group of PLHIV, but once identified, they probably represent the target patient population for interventions aiming at cardiovascular risk reduction.

Only BP values ≥ 140/90 mmHg were rechecked by a qualified nurse or clinical officer in their offices. Since even attended automated BP measurements as used here are lower than conventional office BP values [83], this probably had not introduced a major rate of misclassified truly hypertensive BP categories. 24-h ambulatory BP monitoring (24-h-ABPM) was not available when recruitment for the LighTen Study started. However, when 24-h-ABPM was performed in a subgroup of 117 LighTen participants, a median of 26 months (6–8 visits) after enrollment, white coat hypertension or white coat effect was identified in 18.8% of those with office hypertension [84]. Self-reported hypertension could have been another important source of bias and is known to be considerably flawed [85], but patients without antihypertensive drug treatment and BP values < 140/90 mmHg were not included in the calculation of the prevalence of hypertension. Data on creatinine plasma levels were available only at study entry with no information on albuminuria; thus, the exact KDIGO classification of kidney function [86] was not possible. Also, since the changes in BP and the prevalence and incidence of hypertension were defined as secondary outcomes, the findings of the present study may only be considered as the results of a descriptive data analysis.

We did not address adherence to antihypertensive drug treatment directly, e.g., by pill counting and the inference from viral load suppression as a marker of good adherence may be equivocal. However, PLHIV on ART with undetectable HIV viral load had lower systolic BP among those diagnosed with hypertension [10]. HIV viral replication was suppressed already at study entry in 51 participants (4%); hence, they must have been pretreated, but the type of ART for these patients is unknown. This low number probably has no major impact on the results. Diabetes mellitus type 2 was known in 14 PLHIV with metformin given in 3 cases. Due to the low numbers, these participants were not separately analyzed. Patients were not screened for diabetes, thus, its true prevalence in this cohort is unknown. However, newly diagnosed diabetes needing glucose-lowering drugs was recently reported in only 1 of 100 overweight or obese PLHIV taking ART at the Lighthouse Clinic [87].

## Conclusions

Weight gain during follow-up and initiating ART already at a higher BP level favored a further increase in BP in this cohort of PLHIV. The control rate of hypertension remained low despite increased antihypertensive treatment rates. Our data advocate the inclusion of patient education addressing weight control and antihypertensive treatment adherence as part of NCD management programs at centers caring for PLHIV in low-resources settings like Malawi. Together with intensified training of medical staff to overcome provider inertia in case of indicated treatment adjustments, improved control rates of hypertension might eventually be achieved.

### Supplementary Information

Below is the link to the electronic supplementary material.

Supplementary file1 (DOCX 309 KB)

## Data Availability

The datasets used and analyzed during the current study are available from the corresponding author on reasonable request.

## Abbreviations

AML: Amlodipine
ART: Antiretroviral therapy
ATEN: Atenolol
BMI: Body mass index
BP: Blood pressure
CVD: Cardiovascular disease
DTG: Dolutegravir
EFV: Efavirenz
eGFR: Estimated glomerular filtration rate
ENA: Enalapril
HCTZ: Hydrochlorothiazide
NCD: Non-communicable diseases
NNRTI: Non-nucleoside reverse transcriptase inhibitor
NRTI: Nucleoside reverse transcriptase inhibitors
PLHIV: People living with HIV
3TC: Lamivudine
TDF: Tenofovir disoproxil fumarate
