# Supplementary material for: Blood pressure changes during tenofovir-based antiretroviral therapy among people living with HIV in Lilongwe, Malawi: results from the prospective LighTen Cohort Study
Source: Clin Res Cardiol. 2023 Jul 6;112(11):1650–63. doi: 10.1007/s00392-023-02253-w (PMC10584708; doi:10.1007/s00392-023-02253-w)
Supplement: Supplementary file 1 — Supplementary file1 (DOCX 309 KB) [file 392_2023_2253_MOESM1_ESM.docx]

**Clin Res Cardiol**

**Blood pressure changes during tenofovir-based antiretroviral therapy among people living with HIV in Lilongwe, Malawi – results from the prospective LighTen Cohort Study**

Hans-Michael Steffen, Melani Ratih Mahanani, Florian Neuhann, Angelina Nhlema, Philipp Kasper, Andrew de Forest, Thom Chaweza, Hannock Tweya, Tom Heller, Jane Chiwoko, Volker Winkler, Sam Phiri

*Corresponding author:

Prof. Dr. Hans-Michael Steffen, University Hospital of Cologne,

Clinic for Gastroenterology and Hepatology, Kerpener Str. 62, D-50937 Köln, Germany Tel: +49-221-47886109, Fax: +49-221-47886581.

Email: hans-michael.steffen@uk-koeln.de

**Supplementary information**

Table 1 Characteristics of enrolled at baseline vs. still active at month six vs. not enrolled participants

| **Characteristics** | **enrolled at baseline**  **n=1288** | | **still active at month six**  **n=1135** | | **not enrolled**  **n=1072** | |
| --- | --- | --- | --- | --- | --- | --- |
| Sex |  |  |  |  |  |  |
| Male | 537 | 41.7% | 473 | 41.7% | 481 | 44.9% |
| Female | 751 | 58.3% | 662 | 58.3% | 591 | 55.1% |
| Age at ART start (years)  (mean±SD) | 36.1±9.3 |  | 36.7±9.3 |  | 35.2±9.9 |  |
| BMI (kg/m^2^)  (mean±SD) | 24.4±4.8 |  | 24.9±4.7 |  | 23.5±4.5 |  |
| WHO stage at ART start |  |  |  |  |  |  |
| WHO stage 1 | 603 | 46.8% | 539 | 47.5% | 526 | 49.1% |
| WHO stage 2 | 212 | 16.4% | 188 | 16.5% | 180 | 16.8% |
| WHO stage 3 | 396 | 30.8% | 340 | 30.0% | 271 | 25.3% |
| WHO stage 4 | 77 | 6.0% | 68 | 6.0% | 95 | 8.9% |

Table 2 Follow-up characteristics over visit months

| **After/before visit month** | **Stopped ART** | **Changed ART** | **Lost to follow-up** | **Died** | **Transferred out** | **Withdrew** | **study participants** |
| --- | --- | --- | --- | --- | --- | --- | --- |
| 0 |  |  |  |  |  |  | Baseline n=1,288 |
| 1/3 | 0 | 9 | 33 | 6 | 9 | 8 | -65 |
| 3/6 | 1 | 2 | 30 | 4 | 25 | 6 | -68 |
| 6/12 | 1 | 7 | 41 | 8 | 29 | 6 | -92 |
| 12/18 | 0 | 21 | 43 | 8 | 16 | 3 | -91 |
| 18/24 | 0 | 10 | 29 | 1 | 15 | 2 | -57 |
| 24/30 | 2 | 3 | 24 | 1 | 13 | 1 | -44 |
| 30/36 | 1 | 4 | 21 | 1 | 11 | 1 | -39 |
| 36 |  |  |  |  |  |  | Final n=832 |

Table 3 Changes in body weight of those who dropped out before month 36 vs. those followed until month 36

| **Characteristics** | **Those who dropped out before month 36** | | **Those who visited month 36** | | **p-value** |
| --- | --- | --- | --- | --- | --- |
|  | **Mean** | **SD** | **Mean** | **SD** |  |
| Changes in weight [kg] |  |  |  |  |  |
| Month 0-6 | 1.0 | 4.9 | 1.1 | 4.3 | 0.779 |
| Month 6-18 | 0.4 | 5.1 | 1.4 | 4.1 | 0.021 |
| Month 18-30 | -0.9 | 4.8 | 0.8 | 3.7 | 0.011 |


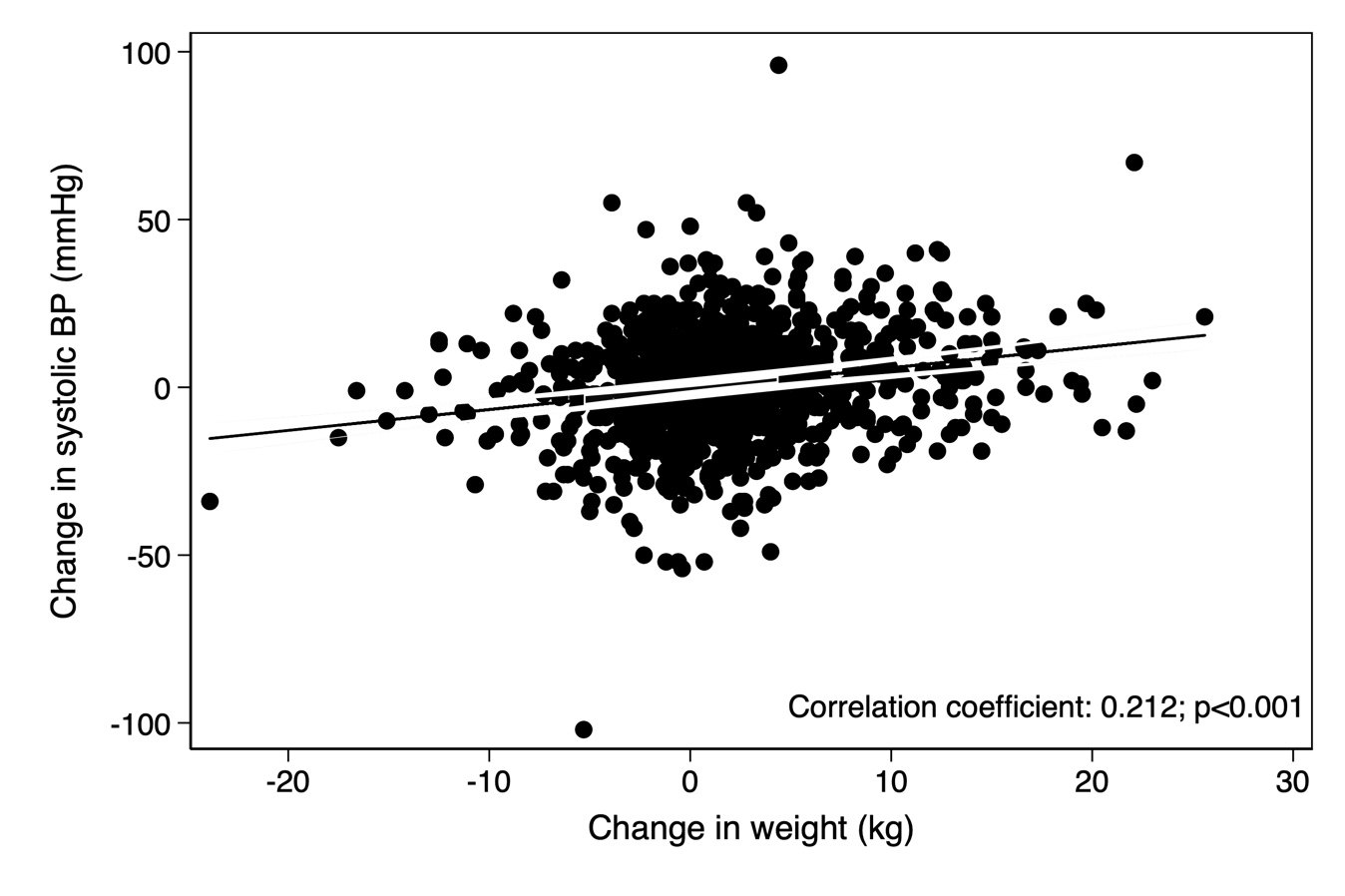


**Fig.1** Observed change in systolic blood pressure (mmHg) and change in weight (kg) from month 6 to each individual’s last visit


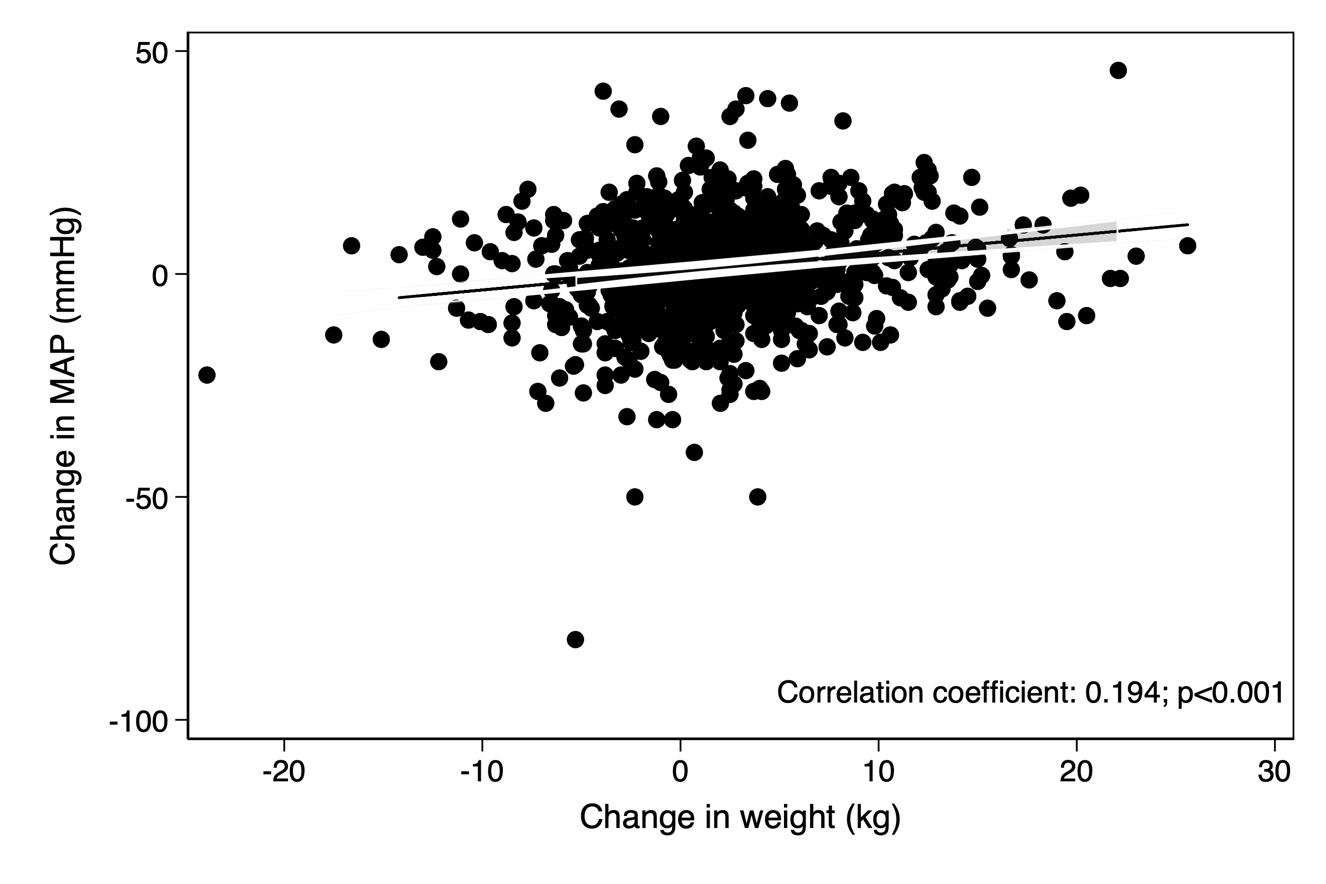


**Fig. 2** Observed change in mean arterial pressure (mmHg) and change in weight (kg) from month 6 to each individual’s last visit
